# Leprosy, Syphilis, Struma

**Published:** 1861-02

**Authors:** 


### Leprosy, Syphilis, Struma.

Dr. Valentine Mott, of the University Medical College, made lately, in one of his lectures, a striking and novel statement. It was to this effect: that to his mind the conviction was irresistible that leprosy was the great progenitor of both syphilis and struma; that they were all three essentially the same disease. His conviction, he stated, was founded upon extensive observations which he had been enabled to make upon leprosy in its various phases, while traveling in the East. The analogy between lepros and syphilitic sore throats and skin diseases he instanced as being particulary striking and complete. He did not enter at large into the subject, but threw out these remarks merely in a suggestive way.—*American Druggist Circular.*

